# Improving Intensive Care Unit Early Readmission Prediction Using Optimized and Explainable Machine Learning

**DOI:** 10.3390/ijerph20043455

**Published:** 2023-02-16

**Authors:** José A. González-Nóvoa, Silvia Campanioni, Laura Busto, José Fariña, Juan J. Rodríguez-Andina, Dolores Vila, Andrés Íñiguez, César Veiga

**Affiliations:** 1 Galicia Sur Health Research Institute (IIS Galicia Sur), Álvaro Cunqueiro Hospital, 36310 Vigo, Spain; 2 Department of Electronic Technology, University of Vigo, 36310 Vigo, Spain; 3 Intensive Care Unit Department, Complexo Hospitalario Universitario de Vigo (SERGAS), Álvaro Cunqueiro Hospital, 36213 Vigo, Spain; 4 Cardiology Department, Complexo Hospitalario Universitario de Vigo (SERGAS), Álvaro Cunqueiro Hospital, 36213 Vigo, Spain

**Keywords:** artificial intelligence, automated machine learning, Bayesian optimization, explainable machine learning, readmission, intensive care unit, machine learning, MIMIC, SHAP, XGBoost

## Abstract

It is of great interest to develop and introduce new techniques to automatically and efficiently analyze the enormous amount of data generated in today’s hospitals, using state-of-the-art artificial intelligence methods. Patients readmitted to the ICU in the same hospital stay have a higher risk of mortality, morbidity, longer length of stay, and increased cost. The methodology proposed to predict ICU readmission could improve the patients’ care. The objective of this work is to explore and evaluate the potential improvement of existing models for predicting early ICU patient readmission by using optimized artificial intelligence algorithms and explainability techniques. In this work, XGBoost is used as a predictor model, combined with Bayesian techniques to optimize it. The results obtained predicted early ICU readmission (AUROC of 0.92 ± 0.03) improves state-of-the-art consulted works (whose AUROC oscillate between 0.66 and 0.78). Moreover, we explain the internal functioning of the model by using Shapley Additive Explanation-based techniques, allowing us to understand the model internal performance and to obtain useful information, as patient-specific information, the thresholds from which a feature begins to be critical for a certain group of patients, and the feature importance ranking.

## 1. Introduction

Readmission to the Intensive Care Unit (ICU) during the same-hospital admission is an uncommon adverse event and could cause a high burden to healthcare systems, with very important socioeconomic effects on patients, relatives and health practitioners [1]. Early and unplanned ICU readmissions, with readmission rates ranging from 1.3% to 13.7% [2], are associated with an increased risk of mortality, morbidity, longer stays in the hospital and ICU, and an increased cost. Consequently, there has been a high interest in the ICU readmission rate as a quality indicator of critical care [2]. Nevertheless, current studies have shown that ICU readmission rates are influenced by factors other than quality of care, such as patient characteristics and length of stay [1], and in general all possible data sources. This opens the problem for the use of new artificial intelligence techniques in order to exploit all the information available.

In recent years, the use of machine learning techniques in the health field has increased in order to improve the patients care quality and to facilitate the health personnel work [3]. Due to the enormous amount of data generated in today’s hospitals, it is of great interest to develop techniques to analyze this data automatically and efficiently, facilitating correct decision-making by healthcare personnel. A manual analysis of all this data would require time that is not available in the day-to-day framework of a hospital [4], leading to only a small portion of it being analyzed, missing the opportunity of analyzing the available data globally. The continuous and exhaustive patients monitoring during their ICU stay produces a wide variety of biomedical data with great potential for applications. The Intensive Care Unit is one of the areas with substantial interest in the application of these techniques [5,6]. Several state-of-the-art articles focus on predicting ICU readmission and quantifying performance through a series of metrics [2]. For example, Barbieri et al. [7] and Rojas et al. [8] obtained an AUROC of 0.74 and 0.76, respectively, both using the MIMIC-III database. Thoral et al. [9] obtained an AUROC of 0.78 using the AmsterdamUMCdb database. Other state-of-the-art consulted works obtained similar results [10,11,12]. In this work, differently than all those above-mentioned papers, we focus on the model’s optimization and its explanation in order to improve the predictions.

The application of artificial intelligence to healthcare involves several ethical concerns, such as unfair algorithmic bias [13,14,15,16]. This is strongly related with the explainability of AI models. In the vast majority of works, predictor models are treated as “black boxes”, without understanding the internal performance and being unable to explain how it reached a certain prediction. This is a problem, especially in critical areas such as healthcare, where ethical aspects are so important. Currently, the field of explainable machine learning is increasing in interest [17], allowing models to be analyzed and to easily perceive, detect, and understand its decision process, i.e., turning them into “white boxes”. Concerning model explainability, Shapley Additive Explanations [18] based on game theory are frequently used. Here are other explanatory techniques in the current state of the art, e.g., based on natural language [19,20]. However, Shapley additive explanation is the only one that satisfies the properties of efficiency, symmetry, dummy and additivity, which together can be considered a definition of a fair payout [21]. Through the use of explicability techniques, information about the model’s internal performance is given: patient-specific information, identifying which features had more weight in the decision; the thresholds from which a feature begins to be critical for a certain group of patients, making it possible to configure alarms that alert healthcare personnel; and the feature importance ranking. This allows us to understand how the model obtains the predictions and to make decisions.

The objective of our work is to explore and evaluate the potential improvement of existing models for predicting ICU patient readmission by using optimized artificial intelligence algorithms and explainability techniques. Specifically, this article analyzes the readmission of patients to the ICU during the same hospital stay. A new methodology based on XGBoost as a predictor model, combined with Bayesian techniques to optimize it, is presented and compared with existing models. Moreover, we explain the internal functioning of the model by using Shapley Additive Explanation-based techniques. As explained above, this prediction is extremely important due to an increased risk of mortality, morbidity, longer stays in hospital and ICU, and an increased cost.

The remainder of the article is structured as follows. In Section 2, the proposed methodology is explained. In Section 3, the results are provided and analyzed. This includes the validation of the ICU readmission prediction model using different statistical metrics as well as explainability outcomes. Finally, the discussion and conclusions of the work are presented.

## 2. Materials and Methods

**Figure 1:**
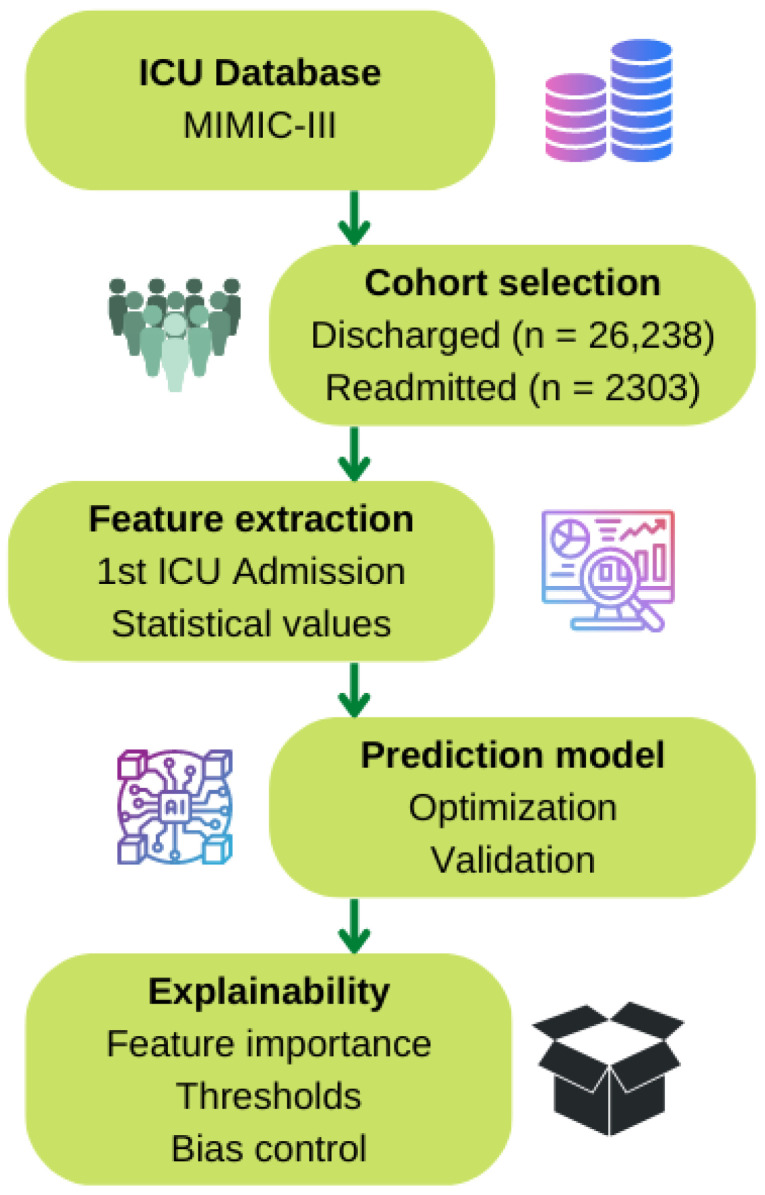
Pipeline of the proposed methodology.

In order to evaluate the benefit of including optimization and explanation stages on the artificial intelligence schema to predict early ICU readmission, a new methodology was developed, which is divided into several stages. The first stage is the cohort selection. The second stage is devoted to extract the features to fit the model. Next, we proceed with the model configuration, both its optimization and validation. Finally, the explainability is performed, extracting the ranking of the most important features, thresholds, and other information of interest. Figure 1 shows the methodology pipeline including all these stages.

### 2.1. Cohort Selection

In this work, the open access database MIMIC-III (Medical Information Mart for Intensive Care III) [22,23] developed by MIT (Massachusetts Institute of Technology) is used to validate the models. It includes information from 61,532 ICU stays at Beth Israel Deaconess Medical Center between 2001 and 2012, such as demographics, vital sign measurements made at the bedside (∼1 data point per hour), laboratory test results, procedures, medications, caregiver notes, or imaging reports, between others. It is available on the Physionet repository [24].

**Figure 2:**
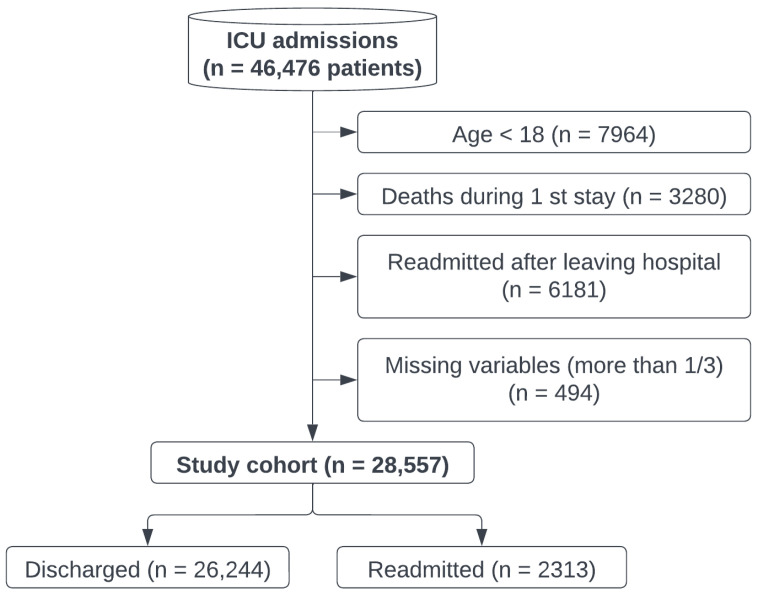
Cohort selection schema.

Regarding the cohort selection, a series of criteria are considered: first, under 18-years-old patients are not included (n = 7964). Those who die during the first ICU stay (n = 3280) are not included in the study either. Moreover, those who were readmitted to the ICU after being discharged from the hospital (n = 6181) are not included. These criteria were followed in other consulted works [2,8,9,10,11,12,25] and will be discussed in detail in Section 4. Finally, patients who do not have measurements of at least 2/3 of the clinical variables that are part of the study are not included (n = 494). A total of 28,557 study patients were obtained, with 2313 patients being readmitted and 26,244 patients not being readmitted. Figure 2 shows the cohort selection schema, and Table 1 shows the patient characteristics for the selected dataset and for the original dataset.

### 2.2. Feature Extraction

The next stage is to extract the features used to feed the predictor model. It is necessary to establish a criterion to determine which clinical variables are used. Following the criteria of other state-of-the-art works [26,27], it was decided to build the models using variables that are present in at least 80% of the patients. A series of statistics (average, standard deviation, minimum and maximum) are extracted from all values collected during the entire first ICU stay. It was also considered to use only the values extracted during the last 24 h of the first ICU stay, but the results obtained were worse, as indicated in Section 4.

Decision trees and ensemble methods, as XGBoost, are not impacted by the outliers in the data, as the data is split by scores that are calculated using the homogeneity of the resultant data points. Consequently, data normalization for feature scaling is not required, as the results are not sensitive to the the variance in the data [28]. Concerning the explicability, data normalization does not affect the results, as the analysis performed is based on the Shapley Additive Techniques, using game theory to iteratively analyze the impact of adding or not adding a feature to the predictor model [21,29].

Table 2 shows the variables used, the features extracted, the mean and standard deviation of each variable, and the measurement units. Except in the case of gender, all features are numeric.

### 2.3. Early-Readmission Predictor Model

There are several approaches in the literature to solve this problem [30]; in this work, it was decided to use the XGBoost model [28], from the family of gradient boosting models. It stands out for being one of the models that obtains the best results in the current state of the art in problems with tabular data [31], in addition to its high efficiency from the computational point of view, supporting the execution in Graphics Processing Units (GPU). In this work, a GPU-based high-performance computing system is used, so the fact that the model can be executed on GPUs is essential to reduce the execution times needed for model optimization.

The variable to predict is the readmission of the patient to the ICU without being discharged from the hospital. As previously indicated, these are the patients who have a higher risk of mortality and longer stays in the ICU. This will be discussed in more detail in Section 4. The model configuration includes both its optimization and validation.

#### 2.3.1. Model Optimization

Regarding the predictor model optimization, this is done both from the computational level and from the prediction quality level. To improve the results of the predictor model, it is necessary to find the best parameters configuration. There are different possibilities to carry out this task [32]. On the one hand, different combinations of parameters can be manually tested, selecting the one that obtains the best results. However, there is usually not a direct relationship between a certain parameter value and prediction quality, but what is important is the combination of different parameter values [33,34,35]. For this reason, the process must be performed automatically. This is part of the research field popularly called Automated Machine Learning. The grid search technique and the random search technique are the most used in the current state of the art. The first consists of testing all parameter combinations without following a certain criterion, while the second is similar to the first with the difference that it does not test all the combinations, using a random search criterion. The first has the disadvantage of being very expensive from a computational point of view, while the second has the disadvantage that it does not follow any criteria searching for the best combination, which does not guarantee that the best combination will be obtained. However, there is a third option, which is used in this work: Bayesian optimization techniques [36]. These, despite being more complex from the conceptual point of view, are characterized by being more efficient in the search. In this work, the Tree-structured Parzen Estimator (TPE) of the open-source Hyperopt package [37] was used, which is based on Bayesian optimization techniques.

**Figure 3:**
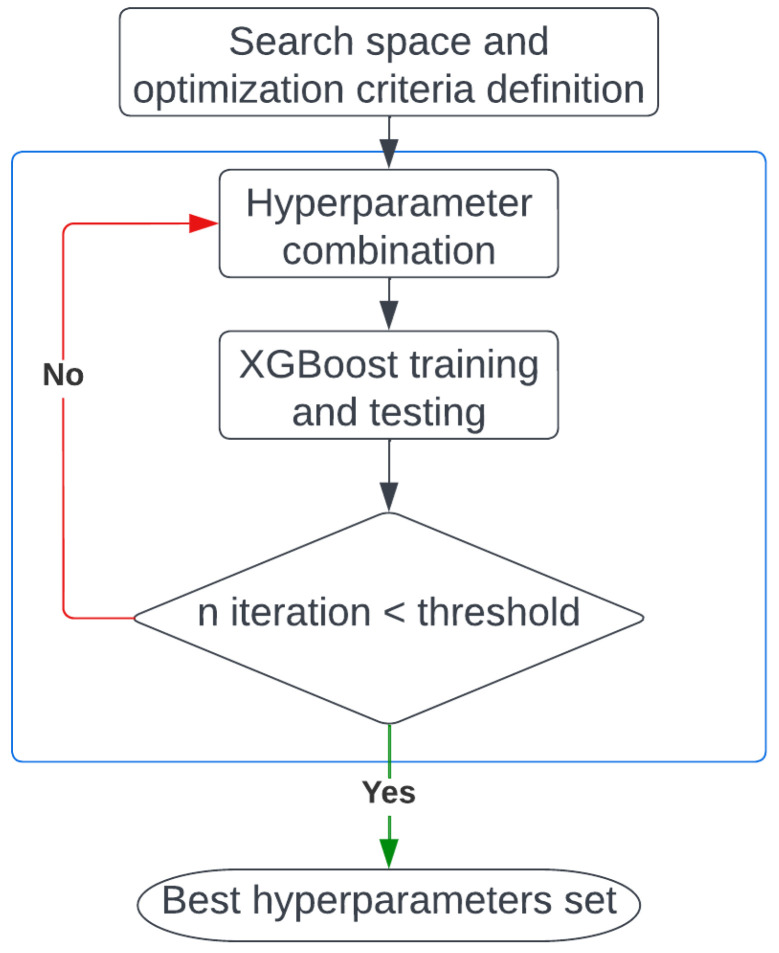
Pipeline of the model hyperparameters optimization.

The first step of this stage is the search space definition, i.e., the hyperparameters value limits between which the TPE will determine the best combination iteratively. Table 3 shows the search space used. The next step is the definition of the optimization criterion used to quantify the predictor model quality. In this work, two different criteria are used: Area Under Receiver Operating Characteristic Curve (AUROC) and Area Under Precision Recall Curve (AUPRC). Table 3 shows the best hyperparameters combination obtained with each criterion. To feed the model, a split training and test is performed, using 70% of the data as training and the remaining 30% as test, shuffling them randomly beforehand. In each iteration, the XGBoost model is trained and tested with the corresponding combination of hyperparameters. Finally, the criterion to consider as completing the optimization process is defined. In this work, the optimization process is finished after 500 search iterations. Figure 3 shows the optimization process pipeline.

#### 2.3.2. Model Validation

The next stage after the model optimization is its validation. The stratified cross-validation method is used to avoid a lucky training–test split, distributing the data in stratified k-folds. Each fold contains approximately the same sample percentage of each target class as the complete set. The number of folds is set to 10. The following metrics are used to validate the model: accuracy (1), specificity (2), F1 score (3), precision (4), recall (5), AUROC and AUPRC, obtained from the confusion matrix, which is shown in Table 4. The metric values obtained are shown in Section 3.

(1) $$Accuracy=\frac{TP+TN}{TP+TN+FP+FN}$$

(2) $$Specificity=\frac{TN}{TN+FP}$$

(3) $$F1=2\times \frac{Precision\times Recall}{Precision+Recall}$$

(4) $$Precision=\frac{TP}{TP+FP}$$

(5) $$Recall=\frac{TP}{TP+FN}$$

## 3. Results

This section presents the results obtained after applying the methodology described in the previous section, relative to the optimization, validation, and explanation stages of the model.

### 3.1. Model Optimization

Using the proposed methodology, it is possible to identify the best set of hyperparameters that provide the best performance in terms of different criteria, as mentioned in Section 2.3.1. Table 3 shows the best XGBoost hyperparameter combination obtained using each of the optimization criteria (AUROC and AUPRC). The results obtained are discussed in Section 4.

### 3.2. Model Validation

**Figure 4:**
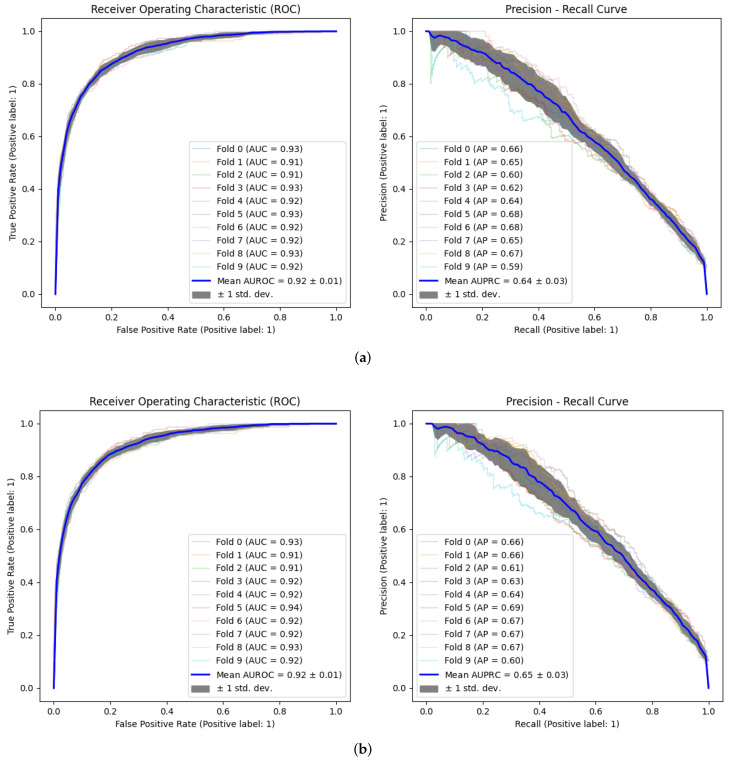
ROC and Precision–Recall curves using the different optimization criteria: (**a**) Using AUROC as optimization criterion. (**b**) Using AUPRC as optimization criterion.

After completing the model optimization stage, we proceed to the model validation. Table 5 shows the different metrics obtained with each hyperparameter combination, compared with the results obtained using the default model configuration. In addition, Figure 4 shows the ROC and Precision–Recall curves, both corresponding to each cross validation step and the average, using the different optimization criteria (AUROC and AUPRC). The values obtained improve the results obtained in the consulted state of the art [7,8,9,10,11,12,25]. Table 6 shows a comparison with related works in terms of AUROC, which is the common metric in most papers that address this problem in the literature. The positive label (1) indicates that admission occurred, while the negative label (0) indicates the patient did not readmit to the ICU. It must be taken into consideration that the values obtained on the referred works have used a different experimental setup than the one proposed in this paper. However, it allows us to define a common base line, as most works use the same database (MIMIC) or the same model (XGBoost).

### 3.3. Explainability

The concept of explainability is related to one of the main problems attributed to the use of artificial intelligence in the healthcare field: using models as “black boxes”, i.e., using a predictive model without knowing how it works internally. The ability to understand the model’s internal performance and be able to explain its behavior is essential, especially in critical areas such as healthcare, where ethical aspects are so important. The explainability of the model allows us to understand how the model obtains the predictions and to make decisions, obtaining useful clinical information: patient-specific information, identifying which features had more weight in the decision; the thresholds from which a feature begins to be critical for a certain group of patients, making it possible to configure alarms that alert healthcare personnel; and the feature importance ranking.

#### 3.3.1. Patient-Specific Information

**Figure 5:**
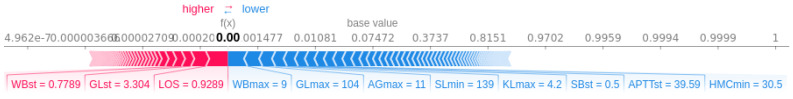
Local explainability of a specific patient outcome prediction.

A useful tool for healthcare personnel is understand the prediction obtained for a specific patient. Figure 5 shows the local explainability of a specific patient, predicted as non-readmission (base value = 0). The features with a higher impact on prediction are closer to the dividing boundary between positive and negative values, and the feature impact is represented by the bar size. Moreover, each feature value is shown next to a feature name. The features in red influence the model to predict a readmission, while the features in blue force the model to predict non-readmission. For example, in this case, the feature length of stay (LOS), with a value of 0.93 days for this patient, impacts the model to predict that patient will be readmitted. On the other hand, the maximum level of white blood cells (WBmax), with a value of 9 × 10^3^ leukocytes, impacts the model to predict that the patient will not be readmitted.

#### 3.3.2. Threshold Identification

**Figure 6:**
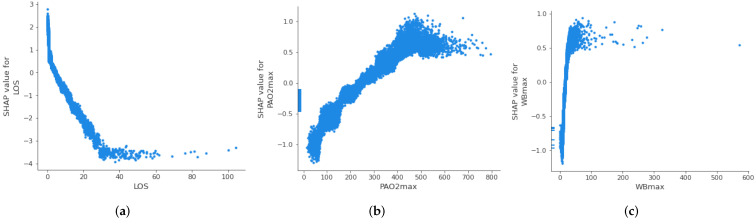
Partial dependence plot of the three more important features: Length of stay (**a**), maximum level of PaO_2_ (**b**), and maximum level of white blood cells (**c**).

Figure 6 shows the relation between feature values and their associated Shapley value. Although only the three most important features are displayed, this analysis could be done for all features. This information is useful to know the thresholds from which the value of a variable begins to be critical for patient health, allowing the definition of alarms and to extract useful clinical information. For example, in the case of PaO_2_, it can be observed that values greater than 200 are related to a greater risk of readmission (SHAP value > 0), while values less than 200 indicate a lower risk of readmission (SHAP value < 0).

#### 3.3.3. Feature Importance

**Figure 7:**
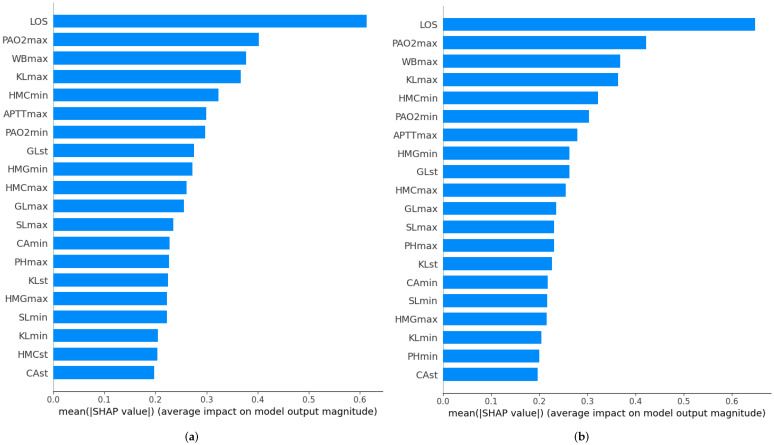
Feature importance using the different optimization criteria: AUROC and AUPRC. (**a**) Using AUROC as an optimization criterion. (**b**) Using AUPRC as an optimization criterion.

Figure 7 shows the feature importance ranking, both using AUROC (Figure 7a) and AUPRC (Figure 7b) as optimization criterion. The Figure x-axis is related to the feature average impact on the model based on the mean Shapley value. The 20 most important features are presented in this paper, the ranking being almost the same in both cases (the top-5 features are exactly the same). The length of ICU stay is the most important feature of the model. However, the ranking of the features does not have to be the same in all patients.

**Figure 8:**
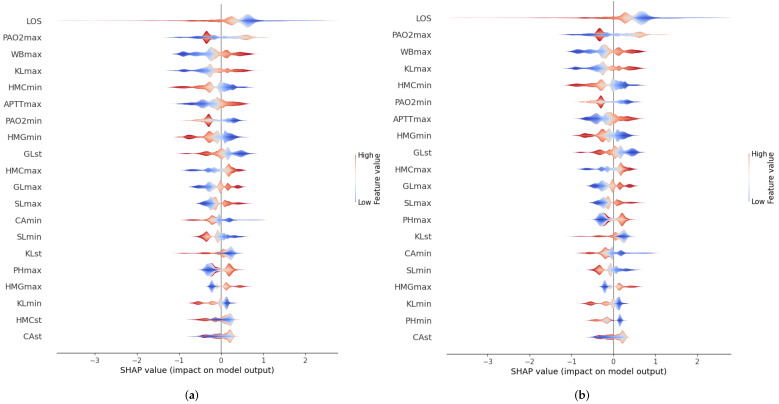
SHAP summary plot using the different optimization criteria: AUROC and AUPRC. (**a**) Using AUROC as an optimization criterion. (**b**) Using AUPRC as an optimization criterion.

In addition to the feature importance ranking, another important element to explain the model performance is to understand how different feature values influence model prediction. Figure 8 shows the Shapley value (abscissas axis) associated with each of the different feature values. The color scale refers to whether the value of the feature is high (red) or low (blue). A feature value with a positive Shapley value associated indicates that it has a positive impact on patient readmission, while a negative Shapley value indicates that it has a positive impact on patient non-readmission. For example, it can be seen that in the case of the length of the ICU stay, higher values influence the model more positively (predicting that the patient has greater chances of readmission) than in the case of lower values.

## 4. Discussion

The results show that a classifier for predicting ICU patient readmission using the methodology described in this work (AUROC = 0.92) outperforms the other state-of-the-art works (measured by AUROC), ranging from 0.66 to 0.81 [2]. For example, Barbieri et al. [7] and Rojas et al. [8] obtained an AUROC of 0.74 and 0.76, respectively, both using the MIMIC-III database. Thoral et al. [9] obtained an AUROC of 0.78, using the AmsterdamUMCdb database. Our results also outperform other previous state-of-the-art consulted works [10,11,12].

The cohort selection and the output variable (patient readmission) are two key elements of the methodology. Regarding the cohort selection, several criteria were used, as indicated in Section 2.1. Patients under 18-years-old were discarded, due to this study being focused on adults ICU. Patients who die during the first ICU stay were also discarded. If they were not discarded, the model will erroneously consider that they do not re-enter the ICU because they were discharged correctly, confusing it. Moreover, this work focused on ICU readmission in the same hospital stay, i.e., without leaving the hospital.

Another option could be to predict ICU readmission regardless of whether it was without leaving the hospital or not. However, there are state-of-the-art works endorsing that patients readmitted to the ICU in the same hospital stay have an increased risk of mortality, morbidity, longer length of hospital and ICU stay, and an increased cost [38]. In addition, ICU patient readmission after leaving the hospital might not be related to the first ICU admission, but rather due to an event that occurred outside the hospital (e.g., an accident). Therefore, patients readmitted to the ICU after leaving the hospital have been also discarded. Finally, patients with more than 1/3 of the missing variables were also discarded. As mentioned in Section 2.2, several statistics extracted from the full 1st ICU stay variables are used as features to feed the model. The effect of extracting the features of the values measured in the last 24 h of the first ICU stay was also analyzed, obtaining worse results (AUROC = 0.69).

During the predictor model optimization process, two different criteria were paralelly used: AUROC and AUPRC. AUROC was used because it is the criterion used in practically all works to compare the results obtained with those of the state-of-the-art. On the other hand, AUPRC was used because it is one of the recommended evaluating criteria to address class-imbalanced data [39]. The results obtained using the different criteria are almost the same, both in relation to the validation of the model and its explainability. In addition, it was proved that the results obtained by optimizing the hyperparameters of the model improve those obtained with the default configuration of this model, as shown in Table 5.

As mentioned above, the application of artificial intelligence to healthcare involves several ethical concerns, such as unfair algorithmic bias [13,14]. In the vast majority of works, predictor models are treated as “black boxes”, without understanding the internal performance and being unable to explain how it reached a certain prediction. In our work, we delved into the internal performance of our model by the use of explainable machine learning techniques, which are currently of broad and current interest. In Section 3.3, some information about the model internal performance was given, including the feature importance ranking and information about how values of each feature impact on prediction. This allows the healthcare personnel and authorities to understand how the model obtains the predictions and to make decisions.

The presented methodology has been validated using the open-access MIMIC-III database. However, the methodology could be applied to another database, being equally valid, or even to other hospital predictors. The differences will be in the intermediate results (the variables that are present in at least 80% of the patients and the cohort number of patients), as well as in the final results (validation metrics obtained).

## 5. Conclusions

This article presents a new methodology to predict early ICU readmission, without being discharged from the hospital, by using artificial intelligence techniques and data collected during the full ICU stay. The predictor model (XGBoost) is optimized to improve the results obtained and validated. Moreover, the model’s internal performance is explained using explainable machine learning techniques.

The results using 28,557 patients demonstrated the validity of the proposed methodology, obtaining an AUROC of 0.92, which improves the state-of-the-art consulted works. The explainability of the model allows us to understand its internal performance and to obtain useful information. This is essential, especially in critical areas such as healthcare, where ethical aspects are so important. In view of the results, it can be concluded that ICU monitoring systems should include optimized and explained artificial intelligence tools.

## Figures and Tables

**Table 1:** Patient characteristics for the selected dataset and for the original dataset.

	MIMIC-III	Cohort
Patients	46,476	28,557
Age (SD ^1^)	55.8 (27.3)	63.3 (18.1)
Gender	M: 26,087 F: 20,380	M: 16,390 F: 12,167
Readmission rate	18.84%	8.10%

^1^ SD: Standard deviation.

**Table 2:** Variables used and features extracted to feed the predictor model.

Variable	Units	Features Extracted	Average	Standard Deviation
Age	Years	Value at 1st admission	63.3	18.1
Gender	-	-	-	-
LOS	Days	-	3.7	5.2
Urine output	mL	Total volume	138.6	3539.4
Glasgow Coma Scale (verbal)	-	Average, standard deviation, maximum, minimum	3.9	1.2
Glasgow Coma Scale (motor)	-		5.6	0.6
Glasgow Coma Scale (eyes)	-		3.6	0.5
Systolic Blood Pressure	mmHg		121.6	15.4
Heart rate	bpm		84.1	13.4
Body temperature	°C		36.8	0.75
PaO_2_	mmHg		165.7	79.7
FiO_2_	mmHg		51.2	11.43
Serum urea nitrogen level	mg/dL		22.1	15.5
White blood cells count	k/uL		10.8	5.7
Serum bicarbonate level	mEq/L		25.5	3.2
Sodium level	mEq/L		138.7	3.3
Potassium level	mEq/L		4.1	0.4
Bilirubin level	mg/dL		1.2	2.8
Breathing Rhythm	bpm		19.3	102.8
Glucose	mg/dL		132.9	42.3
Albumin	g/dL		3.5	5.3
Anion gap	mEq/L		13.2	2.3
Chrolide	mEq/L		105.5	5.9
Creatinine	mg/dL		1.2	1.1
Lactate	mmol/L		2.0	1.1
Calcio	mg/dL		8.5	0.6
Heamotocrit	%		32.2	4.6
Hemoglobin	g/dL		10.97	1.7
International Normalized Ratio (INR)	-		1.4	0.6
Platelets	-		215.8	101.5
Prothrombin Time	s		14.7	3.7
Activated partial thromboplastin time (APTT)	s		35.8	14.1
Base excess	mEq/L		0.1	3.6
PaCO_2_	mmHg		41.84	9.8
PH	-		6.9	0.7
Total CO_2_	mEq/L		25.74	4.3

**Table 3:** Hyperparameters search space and optimal values obtained.

Hyperparameter	Search Space		Optimal Values	
	**Min**	**Max**	**AUROC** **Criterion**	**AUPRC** **Criterion**
Learning rate	−8	0	0.024	0.009
Maximum delta step	0	10	3	4
Maximum depth	1	30	8	23
Maximum n° leaves	0	10	6	8
Minimum child weight	0	15	3	2
N° of estimators	1	10,000	4319	9078
Alpha region	0.1	1	0.912	0.445
Lambda region	0.1	1.5	0.427	0.493
Scale weight	0.1	1	0.851	0.296
Subsample	0.1	1	0.479	0.595

**Table 4:** Confusion matrix.

		Truth (Golden Standard)	
		True	False
Predicted value	True	TP (True Positive)	FP (False Positive)
	False	FN (False Negative)	TN (True Negative)

**Table 5:** Model validation.

	Optimization Criteria		Default Criterion
	AUROC	AUPRC	
AUROC	0.92 (±0.03)	0.92 (±0.02)	0.90 (±0.03)
Accuracy	0.94 (±0.01)	0.94 (±0.01)	0.94 (±0.01)
Specificity	0.99 (±0.01)	0.99 (±0.01)	0.99 (±0.01)
F1	0.53 (±0.12)	0.47 (±0.11)	0.49 (±0.11)
Precision	0.77 (±0.18)	0.85 (±0.17)	0.74 (±0.13)
Recall	0.40 (±0.09)	0.32 (±0.10)	0.37 (±0.10)
AUPRC	0.64 (±0.09)	0.65 (±0.09)	0.60 (±0.10)

**Table 6:** Comparison with related works in terms of AUROC.

Author	Dataset	Predictor	AUROC
Badawi et al. [10]	eICU Research Database	Logistic regression	0.71
Fialho et al. [11]	MIMIC-II	Fuzzy Models	0.72
Frost et al. [12]	Own data	Logistic Regression	0.66
Rojas et al. [8]	MIMIC-III	Gradient Boosting Machine	0.76
Thoral et al. [9]	AmsterdamUMCdb	XGBoost	0.78
Barbieri et al. [7]	MIMIC-III	Neural Network (ODE)	0.71
Our work	MIMIC-III	XGBoost	0.92

## Data Availability

The datasets analyzed during the current study are available in the PhysioNet repository, https://physionet.org/content/mimiciii/1.4/ (accessed on 4 February 2021).

## Abbreviations

The following abbreviations are used in this manuscript:

AUPRC	Area Under Precision-Recall Curve
AUROC	Area Under Curve Receiver Operator Characteristic
ICU	Intensive Care Unit
LOS	Length of Stay
MDPI	Multidisciplinary Digital Publishing Institute
MIMIC	Medical Information Mart for Intensive Care
ROC	Receiver Operator Characteristic
SD	Standard Deviation
TN	True Negatives
TP	True Positives
TPE	Tree Parzen Estimator
